# Nutritional status of children in India: household socio-economic condition as the contextual determinant

**DOI:** 10.1186/1475-9276-9-19

**Published:** 2010-08-11

**Authors:** Barun Kanjilal, Papiya Guha Mazumdar, Moumita Mukherjee, M Hafizur Rahman

**Affiliations:** 1 Institute of Health Management Research (IIHMR), Jaipur, India; 2 Future Health Systems India, Institue of Health Management Research, Kolkata, India; 3 Johns Hopkins Bloomberg School of Public Health, Johns Hopkins University, Baltimore, USA

## Abstract

**Background:**

Despite recent achievement in economic progress in India, the fruit of development has failed to secure a better nutritional status among all children of the country. Growing evidence suggest there exists a socio-economic gradient of childhood malnutrition in India. The present paper is an attempt to measure the extent of socio-economic inequality in chronic childhood malnutrition across major states of India and to realize the role of household socio-economic status (SES) as the contextual determinant of nutritional status of children.

**Methods:**

Using National Family Health Survey-3 data, an attempt is made to estimate socio-economic inequality in childhood stunting at the state level through Concentration Index (CI). Multi-level models; random-coefficient and random-slope are employed to study the impact of SES on long-term nutritional status among children, keeping in view the hierarchical nature of data.

**Main findings:**

Across the states, a disproportionate burden of stunting is observed among the children from poor SES, more so in urban areas. The state having lower prevalence of chronic childhood malnutrition shows much higher burden among the poor. Though a negative correlation (r = -0.603, p < .001) is established between Net State Domestic Product (NSDP) and CI values for stunting; the development indicator is not always linearly correlated with intra-state inequality in malnutrition prevalence. Results from multi-level models however show children from highest SES quintile posses 50 percent better nutritional status than those from the poorest quintile.

**Conclusion:**

In spite of the declining trend of chronic childhood malnutrition in India, the concerns remain for its disproportionate burden on the poor. The socio-economic gradient of long-term nutritional status among children needs special focus, more so in the states where chronic malnutrition among children apparently demonstrates a lower prevalence. The paper calls for state specific policies which are designed and implemented on a priority basis, keeping in view the nature of inequality in childhood malnutrition in the country and its differential characteristics across the states.

## Introduction

Despite recent achievement in economic progress in India [1], the fruit of development has failed to secure a better nutritional status of children in the country [2-5]. India presents a typical scenario of South-Asia, fitting the adage of 'Asian Enigma' [6]; where progress in childhood malnutrition seems to have sunken into an apparent undernutrition trap, lagging far behind the other Asian countries characterized by similar levels of economic development [7-10].

Exhibiting a sluggish declining trend over the past decade and a half, the recent estimate from the National Family Health Survey -3 (NFHS-3)- the unique source for tracking the status of child malnutrition in India [11]- indicates about 46 percent of the children under 5 years of age are moderately to severely underweight (thin for age), 38 percent are moderately to severely stunted (short for age), and approximately 19 percent are moderately to severely wasted (thin for height) [12]. The decline in prevalence however becomes unimpressive with the average levels marked by wide inequality in childhood malnutrition across the states and various socio-economic groups [2,3,13,14]. Growing evidence suggests [13] that in India the gap in prevalence of underweight children among the rich and the poor households is increasing over the years with wide regional differentials. From this specific context, the paper is an attempt to study the specific interplay between household socio-economic conditions and the nutritional status for Indian children (particular in respect to stunting, which is an indicator for long-term nutritional status), considering controls for various other established predictors of the chronic child malnutrition lying at individual, maternal, household and community characteristics.

### Socio-economic inequality in childhood malnutrition: Contextualizing the extent in India

Socio-economic differences in morbidity and mortality rates across the world have received its due attention in the recent years [15-17]. Such differentials in health status in-fact are found pervasive across nations cross-cutting stages of development [18-29]. Studies have identified poverty as the chief determinant of malnutrition in developing countries that perpetuates into intergenerational transfer of poor nutritional status among children and prevents social improvement and equity [30,31]. Nutritional status of under-five children in particular is often considered as one of the most important indicator of a household's living standard and also an important determinant of child survival [32]. The deterministic studies in India while exploring the impact of covariates on the degree of childhood malnutrition suggests an important nexus shared with household socio-economic status [2,25,33-41]. The two-way causality of poverty and under nutrition seems to pose a very significant pretext for malnutrition in India like other developing nations, where poverty and economic insecurity, coupled by constrained access to economic resources permeate malnourishment among the children [42-46]. Thus, economic inequality constitutes the focal point of discussion while studying malnutrition and deserves suitable analytical treatment to examine its interplay with other dimensions of malnutrition and to prioritize appropriate programme intervention. Such attempt to the best of our knowledge is still awaited, using recent nationwide survey data in India.

**Figure 1:**
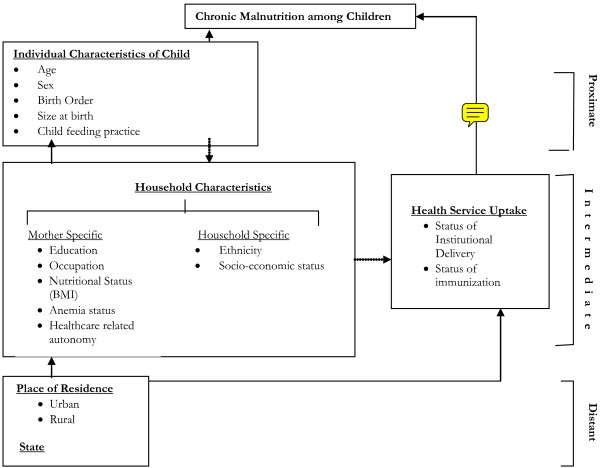
Conceptual Framework

In this backdrop, the paper attempts to shed lights on two specific objectives: 1) to find out the extent of socio-economic inequality in chronic childhood malnutrition, across the major states of India, separated for urban and rural locations, and 2) to understand the conditional impact of household socio-economic condition on nutritional status of children per se, controlling for various other important covariates. The conceptual framework (Figure 1) of the study is based on review of existing literature on the topic and adapts from various existing framework on determinants of childhood malnutrition in general [47], adding a special emphasis on household socio-economic status as the key explanatory variable.

## Methodology

### Data

The paper uses the National Family Health Survey (NFHS) 3rd round data (2005-06) for study and analysis. Similar to NFHS-1 and NFHS-2, NFHS-3 was designed to provide estimates of important maternal and child health indicators including nutritional status for young children (under five years for NFHS-3), following standard anthropometric components. The survey was conducted following stratified sampling technique, details on the sampling procedure can be found at IIPS, 2007 [12]. Of the total 43,737 children for whom NFHS-3 provides height-for-age z-score (HAZ), a subset of 24,896 children was considered; those were alive, hailed from fifteen major states and had the HAZ score within the range of -5 to +5 standard deviation from the WHO-NCHS reference population.

We have also used secondary data from Handbook of Indian Economy 2004-05 [48], for the statistics on per capita Net State Domestic Product (NSDP), for the fifteen major states.

### Methods used

The study uses two analytical methods for studying the objectives. The first objective is catered through the measurement of concentration index and understanding its linkage with the state level indicator of economic development. While for studying the second objective, multi-level regression model have been employed. Further details on methodology are presented below.

### Concentration Index

The widely used standard tool that examines the magnitude of socio-economic inequality in any health outcome, i.e. Concentration Index (CI) [49] is employed to study the extent of inequity in chronic child malnutrition across the states of India. The tool has been universally used by the economists to measure the degree of inequality in various health system indicators, such as health outcome, health care utilization and financing. The value of CI ranges between -1 to +1, hence, if there is no socio-economic differential the value returns zero. A negative value implies that the relevant health variable is concentrated among the poor or disadvantaged people while the opposite is true for its positive values, when poorest are assigned the lowest value of the wealth-index. A zero CI implies a state of horizontal equity which is defined as *equal treatment for equal needs *(For further readings on application of CI in malnutrition refer to Wagstaff & Watanabe 2000) [50]. CI values calculated for stunting help us find the possible concentration among rich and poor children below five years of age during NFHS-3.

### Multi-level regression

Due to the stratified nature of data in NFHS [12], the children are naturally nested into mothers, mothers are nested into households, households are into Primary Sampling Units (PSUs) and PSUs into states. Hence keeping in view this hierarchically clustered nature, the paper uses multi-level regression model to estimate parameter for nutritional status among children to avoid the likely under-estimation of parameters from a single level model [51]. Since here siblings are expected to share certain common characteristics of the mother and the household (mother's education and household economic status for e.g.) and children from a particular community or village have in common community level factors such as availability of health facilities and outcomes, it can be reasonably asserted that unobserved heterogeneity in the outcome variable is also correlated at the cluster levels [52-54]. This amounts to an estimation problem employing conventional OLS estimators, which gives efficient estimates only when the community level covariates and the household level covariates are uncorrelated with the individual and maternal effects covariates.

Researchers have adopted fixed effects models to estimate nutrition models and control for unobservable variables at the cluster level, which leads to the difficulty that if the fixed effect is differenced away, then the effect of those variables that do not vary in a cluster will be lost in the estimation process [54]. Allowing the contextual effects in our analysis of the impact of household socio-economic status on child undernutrition, we adopt an alternative approach of using multilevel models.

Broadly, we test the two types of multilevel models following the practice in contemporary literature; the variance components (or random intercept) models and the random coefficients (or random slopes) models. As in above, STATA routines for hierarchical linear models using maximum likelihood estimators for linear mixed models were used for both model forms.

The variance-components model correct for the problem of correlated observations in a cluster, by introducing a random effect at each cluster. In other words, subjects within the same cluster are allowed to have a shared random intercept. We consider two clusters, i.e., community and household, since in most of the cases NFHS provides information on children of one mother chosen from a particular household. Thus, we have,

$$z_{ij}=\beta \prime x_{ij}+\delta_{i}+\mu_{ij}$$

where **z**_**ij **_is the HAZ score for the child(ren) from the j^th ^household in the i^th ^community. **β **is a vector of regression coefficients corresponding to the effects of fixed covariates **x**_**ij**_, which are the observed characteristics of the child, the household and the community. Where, '**i' **is a random community effect denoting the deviation of community i's mean z-score from the grand mean, '**j' **is a random household effect that represents deviation of household ij's mean z-score from the i^th ^community mean. The error terms **δ**_**i **_and **μ**_**ij **_are assumed to be normally distributed with zero mean and variances σ^2^_c _and σ^2,^_h _respectively. As per our arguments above, these terms are non-zero and estimated by variance components models. To the extent that the greater homogeneity of within-cluster observations is not explained by the observed covariates, σ^2^_c_, and σ^2,^_h _will be larger [55].

To evaluate the appropriateness of the multilevel models, we test whether the variances of the random part are different from zero over households and communities. The resulting estimates from the models can be used to assess the Intra Class Correlation (ICC) i.e., the extent to which child undernutrition is correlated within households and communities, before and after we have accounted for the observed effects of covariates **x**_**ij**_. A significantly different ICC from zero suggests appropriateness of random effect models [54]. The ICC coefficient describes the proportion of variation that is attributable to the higher level source of variation. The correlations between the anthropometric outcomes of children in the same community and in the same family are respectively:

$$\rho c=\sigma^{2}c/(\sigma^{2}c+\sigma^{2}h)$$

Following this, the total variability in the individual HAZ scores can be divided into its two components; variance in children's nutritional status among households within communities, and variance among communities. By including covariates at each level, the variance components models allow to examine the extent to which observed differences in the anthropometric scores are attributable to factors operating at each level. Thus, the variance components model described above introduces a random intercept at each level or cluster assuming a constant effect of each of the covariates (on the outcome) across the clusters.

If additionally, we consider the effect of certain covariates to vary across the clusters (for e.g, differential impact of household socio-economic status or mother's education across households and/or communities), we need to introduce a random effect for the slopes as well, leading to a random coefficients model. Under these assumptions, the covariance of the disturbances, and therefore the total variance at each level depend on the values of the predictors [55].

As mentioned earlier, a subset of 24,896 children have been considered for the analysis from the hierarchically clustered NFHS-3 dataset. Hence, our multilevel models are based on observations on 24,896 children from 18,078 households distributed in 2,440 communities/clusters (PSUs). Inclusion of separate levels for children and mothers were considered not necessary since these were almost unitary to the number of households.

The analysis is presented in the form of five models, apart from the conventional OLS model without considering the cluster random effects, primarily as a comparison: Model_Null is the null model, where the HAZ z scores is the dependant variable with no covariates included; while in the later models along with poorest and richest household asset quintile, other covariates are introduced in a phased manner. Such as, Model_Kids introduces child specific predictors (being purely individual attributes); Model_Moms introduces the mother-specific covariates. Model_Full is the full model with all the model covariates at respective levels. These models are three-level random intercept models with the two clusters: community, and households. In Model_Random_Slope, we introduce a random coefficient for socio-economic status at the household level. We settled for the random coefficient in the form of wealth quintile dummies. The covariates included as controls in our analytical models, with the primary aim of isolating the effect of income or socioeconomic status (SES) on chronic child undernutrition are described below. In the multilevel framework most of these variables can be classified as individual-specific, household-specific or community-specific covariates.

### Variables in the regression model

#### Measuring nutritional status: the dependent variable

As mentioned earlier, the paper uses height for age (stunting) as the key outcome variable, which is an indicator of chronic nutritional status capable of reflecting long-term deprivation of food [56] following the established practice of anthropometric measures of malnutrition. The measure is expressed in the form of z-scores standard deviation (SD) from the median of the 2006 WHO International Reference Population. This continuous standard deviation of HAZ score is capable of providing expected change in the value of the response variable due to one unit change in the regressors regardless of whether a child is stunted or not [57]. Hence, the present approach differs from the usual practice of employing a dichotomous variable on probability of a child being chronically malnourished (0 = otherwise, 1 = stunted). Since here the attempt is not to model probability of stunting, but instead using a deterministic model the paper attempts to find out the influencing role of household asset on childhood nutrition in fifteen major states of India.

### Explanatory variables

#### Asset quintile as the proxy for household socio-economic status

Following the standard approach of assessing economic status of the household [28], the paper uses household asset index provided by the NFHS-3. The survey provides the household wealth index based on thirty-three household characteristics and ownership of household assets using a Principal Component analysis (for details on the methodology refer to IIPS 2007) [12]. In the paper we divided the household index into quintiles based on the asset scores adjusted by sample weights. Separate quintiles were developed for rural and urban areas of each state by using state-specific sample weights, to avoid questions on comparability [28].

### Other explanatory variables used as controls

Apart from the above mentioned asset index, other determinants of childhood malnutrition are chosen based on approaches in literature and presented in the conceptual framework (Figure 1) of the study [47]. We consider certain individual characteristics of child as the proximate covariate of chronic malnutrition. These predisposing factors include child's characteristics similar to other studies, such as, child's age in months, quadratic form of age to eliminate the effect on z-score [38] since there exists non-linearity between age and HAZ, sex of the child, birth order, size of child at birth (as a proxy of birth weight) [57], incidence of recent illness, complete doses of immunization and recommended feeding practice; denoted by exclusive breast feeding for infants below six months of age, introduction of nutritional supplements along with or without breastmilk after six months. In view of information provided by NFHS on child feeding, we considered a child is introduced to supplementary food, wherever the child was reported having given any food-stuff irrespective of its breast feeding status, a day preceding the survey date.

The controls on mother's characteristics includes; years of in terms of education, body mass index (BMI), mothers status of anemia, autonomy for seeking medical help for self [58,59] and place of birth for the child of interest. On the household level, except for asset quintile, controls was included for household ethnicity, since a large number of earlier studies found a significant linkage between scheduled tribe/scheduled caste households and childhood undernutrition [2,14]. Community characteristic is regarded as the distant covariate of child malnutrition in the model and include rural-urban place of residence and state. Keeping in mind the large scale variation in childhood mortality and morbidity, the states are considered for each of the models as controls, or as fixed effects in multilevel models.

## Results

### Extent of socio-economic inequity in childhood stunting

**Figure 2:**
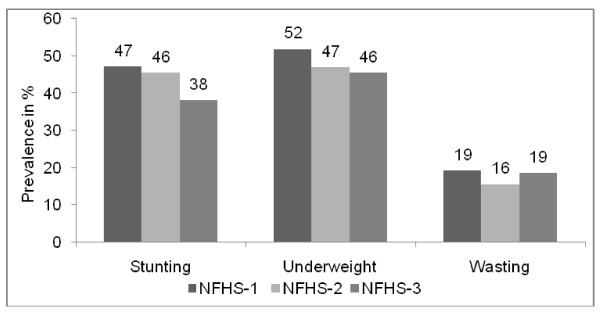
Trend in Malnutrition in India among Children (0-35 months)

As mentioned earlier, the successive waves of NFHS in India indicates a declining trend in the prevalence of child malnutrition among children aged below three years (Figure 2).

Except for wasting, across the two different established anthropometric measures of malnutrition; stunting and underweight, a consistent decline is evident during 1992-2005 period (Figure 2). Overall, NFHS-3 reveals a differential scenario of child malnutrition across the fifteen major states of India (Table 1).

**Table 1:** Prevalence of Malnutrition among children (0-59 months) across fifteen major states of India (NFHS-3)

*States/Country*	*Underweight*			*Stunting*			*Wasting*		
	**Rural**	**Urban**	**Total**	**Rural**	**Urban**	**Total**	**Rural**	**Urban**	**Total**
Haryana	41.3	34.6	39.6	48.1	38.3	45.7	19.7	17.3	19.1
Punjab	26.8	21.4	24.9	37.5	35.1	36.7	9.2	9.2	9.2
Rajasthan	42.5	30.1	39.9	46.3	33.9	43.7	20.3	20.8	20.4
Madhya Pradesh	62.7	51.3	60	51.7	44.3	50	36	31.7	35
Uttar Pradesh	44.1	34.8	42.4	58.4	50.1	56.8	15.2	12.9	14.8
Bihar	57	47.8	55.9	56.5	48.4	55.6	27.4	25.2	27.1
Orissa	42.3	29.7	40.7	46.5	34.9	45.0	20.5	13.4	19.5
West Bengal	42.2	24.7	38.7	48.4	29.3	44.6	17.8	13.5	16.9
Assam	37.7	26.1	36.4	47.8	35.6	46.5	13.6	14.2	13.7
Gujarat	47.9	39.2	44.6	54.8	46.6	51.7	19.9	16.7	18.7
Maharashtra	41.6	30.7	37	49.1	42.3	46.3	18.2	14.1	16.5
Andhra Pradesh	34.8	28	32.5	45.8	36.7	42.7	13	10.7	12.2
Karnataka	41.1	30.7	37.6	47.7	36	43.7	18.2	16.5	17.6
Kerala	26.4	15.4	22.9	25.6	22.2	24.5	18.2	10.9	15.9
Tamilnadu	32.1	27.1	29.8	31.3	30.5	30.9	22.6	21.6	22.2
**All 15 states**	**44.1**	**32.2**	**41.1**	**49.7**	**39.2**	**47.04**	**19.9**	**16.6**	**19.04**

**ALL INDIA**	**45.6**	**32.7**	**42.5**	**50.7**	**39.6**	**48**	**20.7**	**16.9**	**19.8**

Source: Authors' Calculation from NFHS 3 unit data

To describe further, the state of Kerala showed the lowest prevalence of stunting among children (25 percent) across all the major states, where the rural-urban differential is virtually nonexistent. Whereas the opposite side of the spectrum, more than half the children below five years were stunted in Uttar Pradesh (57 percent), Bihar (56 percent), Gujarat (52 percent) and Madhya Pradesh (50 percent) (Table 1). The rural-urban differentials are also considerably high in these states, along with West Bengal; which showed the highest (19 percent) differential between rural-urban prevalence of child malnutrition which is unfavorable for rural areas, during NFHS-3.

Overall, all the three indicators of malnourishment are found highly correlated with each other and hence it was worthwhile to explore their association with the incidence of poverty in the states, following the established line of argument. It can be said that the optimal growth of the children (having standard height for their age and weight for their height) have been strongly associated with economic status of the population.

The CI values for chronic malnutrition in respect to fifteen major states and at the country level consistently return negative values, reflecting a heavy burden of malnutrition among the poor in India (Table 2).

**Table 2:** Concentration Index Values for Stunting across States and Urban-Rural Locations, India, NFHS-3

*States/Country*	*Concentration Index (Stunting)*		
	**Rural**	**Urban**	**Total**
Haryana	-0.118**	-0.257**	-0.151**
Punjab	-0.211**	-0.259**	-0.212**
Rajasthan	-0.069**	-0.182**	-0.106**
Madhya Pradesh	-0.032	-0.133**	-0.063**
Uttar Pradesh	-0.071**	-0.153**	-0.083**
Bihar	-0.082**	-0.131**	-0.094**
Orissa	-0.169**	-0.267**	-0.183**
West Bengal	-0.112**	-0.3**	-0.168**
Assam	-0.101**	-0.253**	-0.116**
Gujarat	-0.087**	-0.132**	-0.115**
Maharashtra	-0.12**	-0.167**	-0.146**
Andhra Pradesh	-0.104**	-0.134**	-0.14**
Karnataka	-0.076**	-0.185**	-0.127**
Kerala	-0.204**	-0.061	-0.165**
Tamil Nadu	-0.075	-0.196**	-0.131**
**All 15 states**	-0.092**	-0.177**	-0.121**

**ALL INDIA**	-0.098**	-0.169**	-0.126**

Source: Authors' Calculation from NFHS 3 unit data

Significant at ***p < 0.01, ** p < 0.05, and * p < 0.10.

The above table (Table 2) confirms the fact that across all fifteen major states and the rural-urban locations, children from poorer households share the higher burden of sub-optimal growth due to undernourishment. It needs special mention that chronic malnutrition among children is more concentrated among urban poor comparing their counterpart living in rural areas. This trend is consistent across all thirteen states, except for Bihar and Kerala; where concentration of stunting is observed higher among poor children from rural areas.

**Figure 3:**
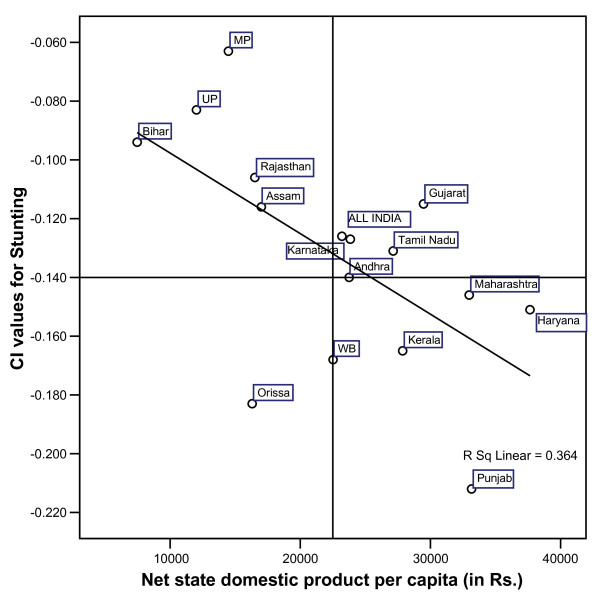
**Scatter plot showing relationship between NSDP and CI for Stunting, across the states**.

It is also seen in a similar vein that aggregate economic status of a population is associated with child nutritional status. CI values for stunting and Net State Domestic Product (NSDP; considered as the indicator for economic development for the aggregate level of the state) share an inverse association (Figure 3), common for most of the states.

Overall, the negative correlation established between CI values for stunting and NSDP per capita stands at r = -0.603 (p < .001). The scatter plot of NSDP per capita and CI values for stunted children across the states (Figure 3) emerges few specific patterns. The states like, Bihar, Uttar Pradesh, Madhya Pradesh and Rajasthan exhibit a typical situation where per capita NSDP is lower than the India average and the burden of malnutrition among the poor is also lesser (indicated by lower negative values of CI). The second group consisting of Gujarat, Tamil Nadu, Karnataka and Andhra located at the north-east quadrant of the scatter diagram, shows a situation where NSDP per capita either equals or surpasses the national average; however a relatively higher burden of chronic malnourishment is found concentrated among the poor. This characteristic is further intensified in the case of Punjab, along with somewhat closer scenario for Kerala and West Bengal and Haryana; where a considerably higher share of stunting can be found among the poor. However, the worst case is noticed for Orissa, where with much lower per-capita NSDP as compared to the national average, the state exhibits a noticeably higher burden of chronic malnourishment among the poor.

### Role of household socio-economic conditions determining long-term nutritional status among children

The results shows (Table 3), significant association between household asset quintiles and nutritional status of children.

**Table 3:** Association (βs) from Ordinary Least Squares and Multilevel Linear Regression Models (Main Effects) between Child Stunting (Height for Age) and Household Socio-Economic Status, controlling for various other covariates; Fifteen Major States, India, NFHS-3

Model parameters	OLS	Null_model	Model_Kids	Model_Mom	Model_Full	Model_Random_Slope
	Exp (B) (SE)					

**HH Asset Quintile**						

Poorest	-0.181*** (0.026)	-	-0.296*** (0.027)	-0.180*** (0.027)	-0.183*** (0.028)	-0.185*** (0.028)
Richest	0.311*** (0.029)	-	0.548*** (0.027)	0.296*** (0.030)	0.314*** (0.031)	0.310*** (0.031)
**Controls**						
*Individual Characteristics*						
Child_age (in months)	-0.085*** (0.002)	-	-0.091*** (0.002)	-0.088*** (0.002)	-0.089*** (0.002)	-0.089*** (0.002)
Child_age2	0.001*** (0.000)	-	0.001*** (0.000)	0.001*** (0.000)	0.001*** (0.000)	0.001*** (0.000)
Sex_male	-0.005 (0.019)	-	-0.012 (0.018)	-0.012 (0.018)	-0.012 (0.018)	-0.012 (0.018)
Order_of_birth	-0.016*** (0.006)	-	-0.041*** (0.006)	-0.018** (0.006)	-0.016** (0.006)	-0.016** (0.006)
Size_at_birth	0.258*** (0.024)	-	0.296*** (0.023)	0.271*** (0.023)	0.265*** (0.024)	0.265*** (0.024)
Suffered_recent_illness	-0.021 (0.026)		-0.018 (0.026)	-0.025 (0.026)	-0.027 (0.027)	-0.026 (0.027)
Recommended_feed	0.042* (0.021)	-	0.043* (0.021)	0.045* (0.021)	0.050* (0.021)	0.050* (0.021)
Completed_immunization	0.007 (0.022)	-	0.090*** (0.022)	0.017 (0.022)	0.014 (0.022)	0.015 (0.022)
*Mothers' characteristics*						
Years_education	0.034*** (0.003)	-	-	0.036*** (0.003)	0.032*** (0.003)	0.031*** (0.003)
BMI_mother	0.026*** (0.003)	-	-	0.029*** (0.003)	0.025*** (0.003)	0.026*** (0.003)
Suffer_anemia	0.113*** (0.025)	-	-	0.104*** (0.026)	0.107*** (0.026)	0.107*** (0.026)
Treatment_self_noprob	-0.054 (0.038)	-	-	-0.046 (0.039)	-0.050 (0.040)	-0.049 (0.040)
Institutional_birth	0.087*** (0.024)	-	-	0.112*** (0.024)	0.075** (0.025)	0.075*** (0.025)
*Household characteristics*						
Ethnicity_SCST	-0.104*** (0.022)	-	-	-	-0.120*** (0.024)	-0.121*** (0.024)
Rural_residence	-0.135*** (0.023)	-	-	-	-0.147*** (0.026)	-0.145*** (0.026)
*States*						
Haryana	-0.129* (0.065)	-	-0.184* (0.083)	-0.103 (0.078)	-0.122 (0.078)	-0.120 (0.078)
Rajasthan	0.161*** (0.060)	-	0.093 (0.077)	0.187** (0.072)	0.160* (0.073)	0.162* (0.073)
Uttar Pradesh	-0.255*** (0.052)	-	-0.237*** (0.065)	-0.215*** (0.062)	-0.259*** (0.062)	-0.258*** (0.062)
Bihar	-0.165*** (0.060)	-	-0.179** (0.077)	-0.115 (0.072)	-0.167* (0.073)	-0.164* (0.073)
Assam	0.131 (0.071)	-	0.115 (0.082)	0.126 (0.077)	0.153 (0.083)	0.153 (0.083)
West Bengal	0.142* (0.060)		0.209** (0.072)	0.186** 0.068)	0.162* (0.070	0.165*** (0.070)
Orissa	0.116 (0.062)	-	0.126 (0.077)	0.123 (0.073)	0.127 (0.073)	0.134 (0.074)
Madhya Pradesh	0.025 (0.056)	-	0.062 (0.071)	0.069 (0.067)	0.032 (0.067)	0.034 (0.067)
Gujarat	-0.307*** (0.063)	-	-0.281*** (0.079)	-0.272*** (0.074)	-0.314*** (0.075)	-0.311*** (0.075)
Maharashtra	-0.161*** (0.058)	-	-0.012 (0.070)	-0.101 (0.067)	-0.156* (0.068)	-0.153* (0.068)
Andhra Pradesh	-0.013 (0.060)	-	0.103 (0.074)	0.041 (0.070)	-0.010	-0.005 (0.070)
Karnataka	-0.031 (0.063)	-	0.074 (0.076)	0.027 (0.073)	-0.002	0.001 (0.074)
Kerala	-0.021 (0.072)		0.229** (0.083)	0.031 (0.079)	-0.005	0.000 (0.082)
Tamil Nadu	0.377*** (0.062)	-	0.54*** (0.075)	0.405*** (0.071)	0.385*** (0.072)	0.385*** (0.072)
_cons	-1.291*** (0.095)	1.593*** (0.014)	-0.184*** (0.083)	-1.471*** (0.094)	-1.200*** (0.102)	-1.208*** (0.102)

**N**	**23099**	**24896**	**24541**	**23939**	**23099**	**23099**
- 2 log likelihood	(R^2 ^0.170)	-45882.64	-43383.54	-42042.3	-40559.133	-40553.034
***X***^***2***^statistics	152.390	-	4123.31	4657.03	4575.07	4527.58
P value	0.000	-	0.000	0.000	0.000	0.000

Significant at *** p < .001, ** p <.01, *p < .05

Given the form of the dependent variable in the subsequent models a higher coefficient indicates better nutritional status among children from better off socio-economic status quintiles (SES). It shows (Table 3) nearly 50 percent better nutritional status (0.31 - (-0.18)) among children from richest SES quintiles, compared to ones those from the poorest quintile.

The variance component models (i.e., Model_kids, Model_moms and Model_full) and the random slope model (Table 3) also support such finding. By introducing covariates at each level, the variance component models allow to examine the extent to which observed differences in the HAZ scores are attributed to the factors operating at each level. With the introduction of child's individual characteristics in the Model_kids along with the state level fixed effect, the impact of richest & poorest SES quintiles become much stronger. The result shows over 80 percent (0.54 - (-0.29)) higher incidence of worse nutritional status among children in the poorest quintile, than the ones hailing from richest SES group. However, such richest-poorest gap decreases with the phased introduction of covariates related to mother's characteristics, household ethnicity and place of residence in the models (Table 3). Finally, similar to the initial estimate by OLS, the variance component models and random slope model indicates that the children with the most favorable SES background enjoy almost 50 percent better nutritional status than their counterpart from the poorest SES groups.

The calculated ICC coefficient values presented in Table 4 differ from zero. This indicates that child nutrition is indeed correlated with households and communities (PSUs). The ICC for household level shows much higher correlation than the case of PSUs.

**Table 4:** Random Coefficients, Intra-class correlation and Variance Decomposition estimates from comparative models

	Null_model	Model_Kids	Model_Mom	Model_Full	Model_Random_Slope
	**Random Effects**				
**σ**^**2**^**_c _(Community - PSU)**	0.202	0.091	0.056	0.055	0.055
(S.E.)	(0.014)	(0.009)	(0.008)	(0.008)	(0.008)
Proportions of overall (null model) explained by the covariates of the model (in %)		55.189	72.149	72.850	72.875
** σ**^**2**^**_h _(Household)**	0.346	0.462	0.436	0.431	0.366
(S.E.)	(0.027)	(0.025)	(0.025)	(0.025)	(0.030)
Proportions of overall (null model) explained by the covariates of the model (in %)		-33.578	-26.086	-24.575	-5.683
Residual^2^	1.877	1.516	1.514	1.518	1.516
(S.E.)	(0.029)	(0.024)	(0.025)	(0.025)	(0.025)
	**Intra-class correlation**				
ρ (PSU)	0.083*	0.044*	0.028*	0.027*	0.028*
ρ (household)	0.226*	0.267*	0.245*	0.242*	0.217*
	**Variance Decomposition (in %)**				
PSU	36.9	16.4	11.4	11.3	13.1
Household	63.1	83.6	88.6	88.7	86.9

Significance level: * *p < 05*

The lower panel of the Table 4 shows how the residual variance is distributed across PSUs and households. Estimates from model 1(null model), which contains no observed covariates, indicate that the variation in height-for-age has substantial group level components. The total variance 0.548 (combined for PSU and households estimates), of which 63 percent is attributed to household level variation in anthropometric scores. Consistent with this observation on null model variance decomposition, other model specifications show similar variance distribution pattern across state, PSU and household levels.

Estimation of household random effects (Table 4) indicates that household heterogeneity is accounted for only partially by the covariates in our model (Model_Full & Model_random_slope). In other words the significantly different values of **σ**^**2**^**_c _**and **σ**^**2**^**_h _**indicates that the homogeneity within cluster observations is not explained by the observed covariates specified in the model. The intra-household correlation remains as large as 0.242 suggesting that the height outcomes of two children belonging to the same family are more homogenous than those of two children chosen at random, even after adjusting for other observed covariates (Model_Full).

Overall, the higher value of **σ**^**2**^**_h _**; i.e., variance at household level denotes existence of higher homogeneity at the household level. These results further imply that choice of one-level model with the similar data set might yield underestimation of parameters.

## Discussion

Successive waves of NFHS brings to the fore widespread under nutrition among the Indian children, however it shows a declining trend during the inter survey period. Though, the latest estimates as provided by the NFHS 3, highlights the continuance of high overall levels of child malnutrition in India. As we find here, prevalence of child malnutrition in India is widely varied across the states and also across rural and urban areas. It needs special mention that chronic malnutrition among children is more concentrated among urban poor compared to their counterpart living in rural areas (Table 2) where inequalities are not as great but overall levels of malnutrition are higher. This trend is consistent across all thirteen states, except for Bihar and Kerala; where concentration of stunting is observed higher among poor children from rural areas.

The intra-state inequality in child malnutrition is stark as we find through the divergent values of the Concentration Index highlighting the disproportionate burden among the poor. The variance component models clearly show clustering of observation at community and household levels. In other words, for the fifteen major states in India, children in the households that shared similar communities do posses similar nutritional status. Intra-household correlation is the most substantial, comparing intra-PSU correlation. In other words, children from a cluster or community do not seem to share stronger correlation in terms of their nutritional status. But, at the household level the observations are not independent. It implies the fact that children belonging to a particular household do share certain common characteristics while growing up. The children who belong to households from the poorest SES quintile have higher prevalence of worse nutritional status. While, on the contrary the children hailing from richest asset quintile households are associated with better nutritional status. The finding is supportive of many earlier observations made based on NFHS data [2]. Such association is consistent across the different models applied to the research (Table 4); reconfirming better nutritional status among children with favourable household socio-economic background, even after controlling for a range of individual, maternal and community characteristics. This further emphasizes the impact of differential available resources to the families that act as a major determinant of malnutrition. The finding is supportive of studies conducted even in other countries [60]. Hence the gradient of household socio-economic status remains as a crucial determinant of level of nutritional achievement among children. Betterment of such condition thus is expected to improve growth of children likely through better nutritional intake and reduced morbidity.

However, at the more macro level it is seen that absolute levels of malnutrition prevalence across the states is not necessarily linearly correlated with the intra-state inequality in malnutrition prevalence. In other words, states that records higher prevalence of childhood malnutrition are not always reflective of the disproportionate burden shared by the poorest households. Mazumdar (forthcoming) [61], while exploring the linkage between poverty and inequality with child malnutrition in India suggests a possible conformation of malnutrition inequality with overall socioeconomic inequality that exists in the states. We too identify a similar pattern; though with overall economic development measured through NSDP is found to be negatively correlated with the proportion of stunted children in the state, emphasizing the role of development that promotes equity in better nutritional outcome; the pattern cannot be generalized.

In states like Punjab and Kerala with better development, a typical scenario emerges. Here, higher inequality in malnutrition prevalence can be observed at the lower levels of percentage of stunted children. On the other hand, states like Madhya Pradesh, Bihar, Gujarat and Rajasthan the states with less economic development or at par with the national average, though have considerably high prevalence of malnutrition exhibited lower values of the concentration index suggesting lower levels of inequality. It is particularly since a higher average implies prevalence of malnutrition irrespective of SES with fewer differentials. Hence a clear gradient of malnutrition inequality, biased against the poor is more pronounced in states where absolute levels of malnutrition are low. This is largely due to the overall inequality in household asset [50,62] among the states, with the poor accounting for a major share of the malnourished children.

On the other hand, the states with higher levels of child malnutrition, generally tend to have a uniform distribution of malnourished children across the socio-economic distribution (Mazumdar *forthcoming*) [61], and the poor in states with lower observed levels sharing a higher disproportionate burden, vis-à-vis the poor in the former group of states.

The situation in Orissa is however the worst and does not confirm to any of the pattern discussed above. Here, with much lower per-capita NSDP as compared to the national average, the state exhibits a noticeably higher burden of chronic malnourishment among the poor. Hence, perhaps economic development cannot be considered as the straightforward indicator for removing overall disparity in various input and outcome indicators among different income bracket, especially in a country like India. It is argued that reduction in child malnutrition does not seem to depend so much on economic growth of a state per se or even on the efforts at reducing income poverty at the state level [3]. Achieving better nutritional status among children is found sharing close nexus with the household socio-economic conditions, efforts to influence households' economic status thus might prove to be beneficial. Alternatively, one can only think of successful targeted interventions to ensure nutritional status among children those belong to unfavorable asset bracket.

Nevertheless, this issue of malnutrition and poverty deserves special treatment incorporating other parameters reflecting the possible predictors of overall socioeconomic inequality and its bearing on malnutrition inequality among the states, as future research in this area. Attempts can be worthwhile to know the reasons why the states with better economic development coupled with noticeable success in arresting the overall level of chronic child malnutrition, have failed to remove its disproportionate prevalence across the socio-economic classes. It can be said that prevalence of worse nutritional status among children in India cannot be addressed with utmost success unless, inequality in prevalence across socio-economic classes are taken care of. A more state specific policy should be designed on a priority basis, to arrest such unequal prevalence.

## Conclusions

Regional heterogeneity in malnutrition across the major states and rural-urban locations are observed to be widespread during NFHS-3. The concerns amplify with the disproportionate burden of malnutrition among poor, more so in the states where the absolute level of malnutrition is seen to be at the lower level, but where they are experiencing better status of economic development. Multilevel analyses with introduction of controls on various covariates continue to indicate the household SES-undernutrition gradient. Hence, an appropriate policy guideline that focuses on altering the nutritional intake among the poor children, especially in the states with apparent lower prevalence of childhood malnutrition is need of the hour. In the high prevalence states much stronger programme are awaited to reduce the overall level. More focused programme attention targeted at the poor to enhance the level of nutrition and behavioral changes, through interventions like the positive deviance approach in a state like Orissa should be further expanded in the near future.

## Competing interests

The authors declare that they have no competing interests.

## Authors' contributions

BK conceptualized, developed the theoretical ideas and oversaw the project. PGM performed analysis and wrote the manuscript. MM contributed to the analysis and helped to draft the manuscript. MHR contributed in theoretical ideas and presentation of the manuscript. All authors read and approved the final manuscript.
